# Fucoidan Extract Induces Apoptosis in MCF-7 Cells via a Mechanism Involving the ROS-Dependent JNK Activation and Mitochondria-Mediated Pathways

**DOI:** 10.1371/journal.pone.0027441

**Published:** 2011-11-11

**Authors:** Zhongyuan Zhang, Kiichiro Teruya, Hiroshi Eto, Sanetaka Shirahata

**Affiliations:** 1 Graduate School of Bioresource and Bioenvironmental Sciences, Kyushu University, Fukuoka, Japan; 2 Faculty of Agriculture, Kyushu University, Fukuoka, Japan; 3 Daiichi Sangyo Co., Ltd., Osaka, Japan; Enzo Life Sciences, Inc., United States of America

## Abstract

**Background:**

Fucoidan extract (FE), an enzymatically digested compound with a low molecular weight, is extracted from brown seaweed. As a natural compound with various actions, FE is attractive, especially in Asian countries, for improving the therapeutic efficacy and safety of cancer treatment. The present study was carried out to investigate the anti-tumor properties of FE in human carcinoma cells and further examine the underlying mechanisms of its activities.

**Methodology/Principal Finding:**

FE inhibits the growth of MCF-7, MDA-MB-231, HeLa, and HT1080 cells. FE-mediated apoptosis in MCF-7 cancer cells is accompanied by DNA fragmentation, nuclear condensation, and phosphatidylserine exposure. FE induces mitochondrial membrane permeabilization (MMP) through loss of mitochondrial membrane potential (ΔΨm) and regulation of the expression of Bcl-2 family members. Release of apoptosis-inducing factor (AIF) and cytochrome *c* precedes MMP. AIF release causes DNA fragmentation, the final stage of apoptosis, via a caspase-independent mitochondrial pathway. Additionally, FE was found to induce phosphorylation of c-Jun N-terminal kinase (JNK), p38, and extracellular signal-regulated kinase (ERK) 1/2, and apoptosis was found to be attenuated by inhibition of JNK. Furthermore, FE-mediated apoptosis was found to involve the generation of reactive oxygen species (ROS), which are responsible for the decrease of ΔΨm and phosphorylation of JNK, p38, and ERK1/2 kinases.

**Conclusions/Significance:**

These data suggest that FE activates a caspase-independent apoptotic pathway in MCF-7 cancer cells through activation of ROS-mediated MAP kinases and regulation of the Bcl-2 family protein-mediated mitochondrial pathway. They also provide evidence that FE deserves further investigation as a natural anticancer and cancer preventive agent.

## Introduction

The polysaccharide known as fucoidan is extracted from marine brown algae and is known to contain large proportions of l-fucose and sulfate, along with low amounts of xylose, uronic acid, and galactose [1]–[2]. Fucoidan has been reported to possess antioxidant, antiviral, antibacterial, anti-inflammatory, and anticoagulant activities [1]–[3]. There is accumulating evidence to support the proposal that the use of fucoidan as a supplement provides protection against various cancers. Clinical trials of patients with breast, cervical, renal, and hepatic carcinomas showed a significant improvement in tumor regression among patients who received an alternative medicine treatment regimen based mainly on fucoidan administration [4]. *In vivo* studies performed using mouse xenograft models have demonstrated that FE suppresses tumor growth of A20-derived lymphoma [5], inhibits metastasis of Lewis lung adenocarcinoma [6] and 13762 MAT rat mammary adenocarcinoma [7], and has anti-angiogenesis activity against Lewis lung adenocarcinoma and B16 melanoma [8]. *In vitro*, several mechanisms have been postulated to underlie the anticancer activity of fucoidan, including induction of apoptosis in cells derived from human lymphoma, promyelocytic leukemia, colon carcinoma, breast carcinoma, and hepatoma and prevention of angiogenesis and invasion in HT1080 fibrosarcoma cells [9]–[15]. However, the molecular mechanisms involved in the anticancer action of fucoidan are complex, and the targets and molecular mechanisms by which it initiates death of cancer cells are incompletely understood.

Apoptosis is a representative form of programmed cell death, which has been assumed to be critical for cancer prevention [16]. Apoptotic cell death is characterized by the activation of either the extrinsic pathway, which is initiated by activation of death receptors leading to cleavage of caspase-8, or the intrinsic pathway, which is marked by mitochondrial depolarization, release of cytochrome *c*, and subsequent activation of caspase-9 [17]–[19]. Although caspase activation is considered a hallmark of apoptotic cell death, other apoptogenic proteins that appear not to require caspase activation have been described. Examples include apoptosis-inducing factor (AIF), endonuclease G, and smac. AIF is a mitochondrial protein that mediates caspase-independent apoptosis in a number of model systems after translocation into the cytosol or into the nucleus [20]–[22]. When AIF is released from the mitochondria in response to apoptotic stimuli, it becomes an active executioner of the cells by causing condensation of chromatin in the nuclei and large-scale fragmentation of DNA [23].

Mitochondria play a central role in the induction and control of apoptosis. The Bcl-2 family members are key players in the mitochondria-dependent intrinsic pathway of apoptosis [24]. The Bcl-2 family consists of pro- and anti-apoptotic proteins that work together and with other proteins to maintain a dynamic balance between the survival and death of cells. The mitogen-activated protein (MAP) kinases, a family of serine/threonine kinases, are also involved in apoptosis and cell survival and have been validated as targets for anticancer drugs [25]–[26]. JNK and p38 are induced by stress responses and cytokines and can mediate differentiation and cell death [25], [27]. ERK1/2 is generally associated with cell proliferation and growth [25], [27]. Previous studies have demonstrated that JNK, p38, and ERK 1/2 have essential roles in modulating the function of the mitochondrial pro- and anti-apoptotic proteins [28]–[31]. ROS are the byproducts of normal cellular oxidative processes and have been suggested to be involved in regulating the initiation of apoptotic signaling. Increased levels of ROS have been demonstrated to induce depolarization of the mitochondrial membrane, which eventually produces an increase in the level of other pro-apoptotic molecules in cells [32]–[33].

On the basis of this knowledge, we determined the growth inhibition activity of FE in several cancer cell lines and further investigated the mechanisms of apoptosis triggered by FE in MCF-7 cancer cells. We have examined activation of members of the caspase family as well as expression of mitochondrial-dependent apoptotic factors and ROS-dependent phosphorylation of MAP kinases in response to FE. Our results indicate that FE induces apoptosis via a caspase-independent pathway involving mitochondrial permeabilization, activation of Bcl-2 family proteins, and release of cytochrome *c* and AIF. ROS-dependent JNK phosphorylation is an upstream cellular event that is involved in the mitochondria-dependent apoptotic pathway.

## Results

### Effect of FE on the growth of cancer cell lines

**Figure 1:**
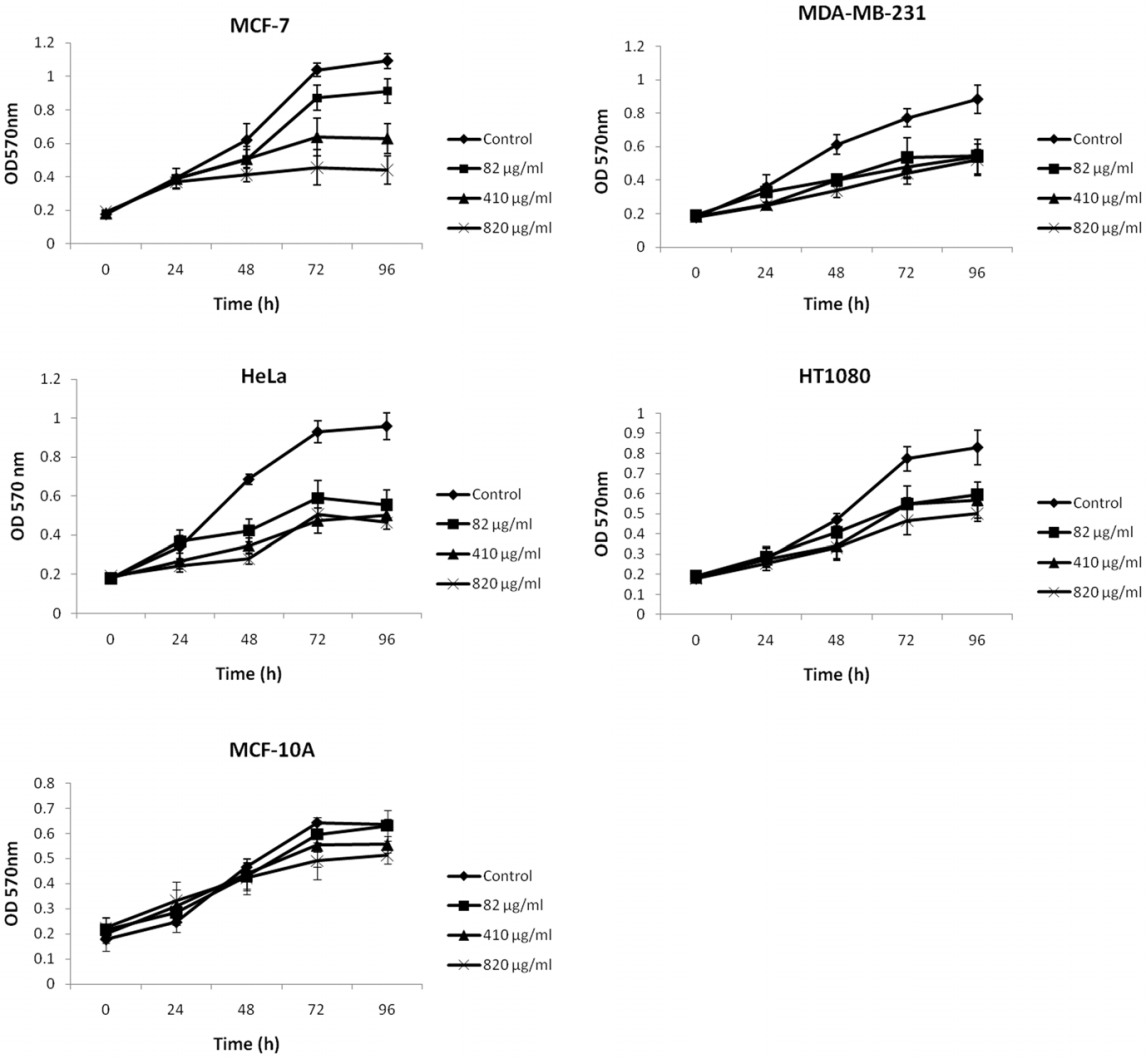
Effect of FE on the growth of cancer cell lines. Cell proliferation was analyzed using the MTT assay. Cells were treated with 82, 410, or 820 µg/mL FE or with PBS as the vehicle control for different time periods (n = 6). Results represent the means ± SD from three independent experiments.

The effects of FE on several carcinoma cell lines were examined using the MTT assay. The cells were treated with different doses of FE for the indicated time periods. As shown in Figure 1, FE treatment inhibits cell growth of MCF-7 (60% growth inhibition after FE treatment for 96 h), MDA-MB-231 (41% growth inhibition after FE treatment for 96 h), HeLa (52% growth inhibition after FE treatment for 96 h), and HT1080 (40% growth inhibition after FE treatment for 96 h) cells. MCF-7 cells were much more sensitive than the other three cell lines to different doses of FE. The non-malignant MCF-10A cell line exhibits lower sensitivity to FE treatment than the malignant cell lines. K562 leukemia, U937 lymphoma, and HL-60 leukemia cell lines have previously been found to be sensitive to growth inhibition by FE treatment (data not shown). These data indicate that FE mediates broad-spectrum growth inhibition of human carcinoma cells.

### FE induces apoptosis in MCF-7 cancer cells

**Figure 2:**
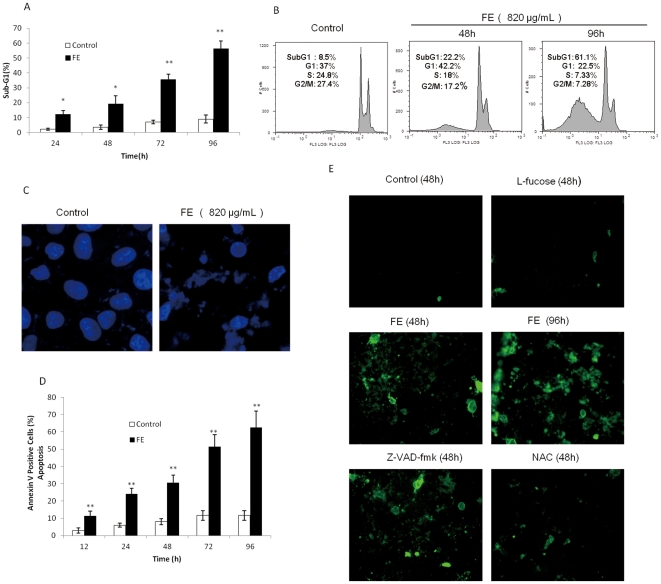
FE induces apoptosis in MCF-7 cancer cells. (A and B) Sub-G1 cell population in control and FE-treated (820 µg/mL) MCF-7 cells was measured by flow cytometry as described in the methods section. The cell distribution was analyzed by flow cytometry analysis software. (C) Hoechst 33342 staining of MCF-7 cells treated with or without 820 µg/mL FE for 96 h. Each experiment shown is representative of 20 random fields observed. (D) Analysis of apoptotic cells by annexin/PI double staining using the IN Cell Analyzer 1000. MCF-7 cells were treated for different time periods with or without 820 µg/mL FE. Apoptosis was evaluated by determining the percentage of annexin V-positive cells. (E) Annexin/PI double staining of MCF-7 cells pretreated with 10 µM Z-VAD-fmk or 2 mM NAC. After a 1-h incubation, cells were exposed to 820 µg/mL FE for the indicated time periods. Cells grown in a medium containing 1 mg/mL L-fucose without FE served as a sugar control. Each experiment shown is representative of 20 random fields observed. All of the experiments were performed at least in triplicate. All results were obtained from three independent experiments. A significant difference between treatment and control groups is indicated by p<0.05(*) or p<0.01(**).

To determine whether FE-induced cytotoxicity involves alterations in cell cycle progression, the DNA content was analyzed by flow cytometry. Figure 2A shows that FE causes a significant increase in the percentage of cells in the sub-G1 phase of the cell cycle in a time-dependent manner. The percentage of cells in the sub-G1 phase increased almost 6-fold over 96 h in the presence of 820 µg/mL FE relative to the control. However, no significant changes in cell cycle distribution were observed (Figure 2B).

The induction of apoptosis by FE in MCF-7 cells was confirmed in fluorescence photomicrographs of cells stained with Hoechst 33342. Figure 2C clearly demonstrates a shrunken nucleus and peripherally clumped and fragmented chromatin. These characteristics are typical of apoptotic cells. FE treatment also caused cells to lose their phospholipid membrane asymmetry. The exposure of phosphatidylserine to the outside of the plasma membranes was detected by annexin V-FITC staining in MCF-7 cells. The annexin V staining results (Figure 2D and 2E) indicate that FE causes a significant increase in the number of apoptotic cells (annexin V positive) in a time-dependent manner. The effect of L-fucose (1 mg/mL) on MCF-7 cancer cells was not significant (Figure 2E).

### FE mediated apoptosis is caspase-independent

**Figure 3:**
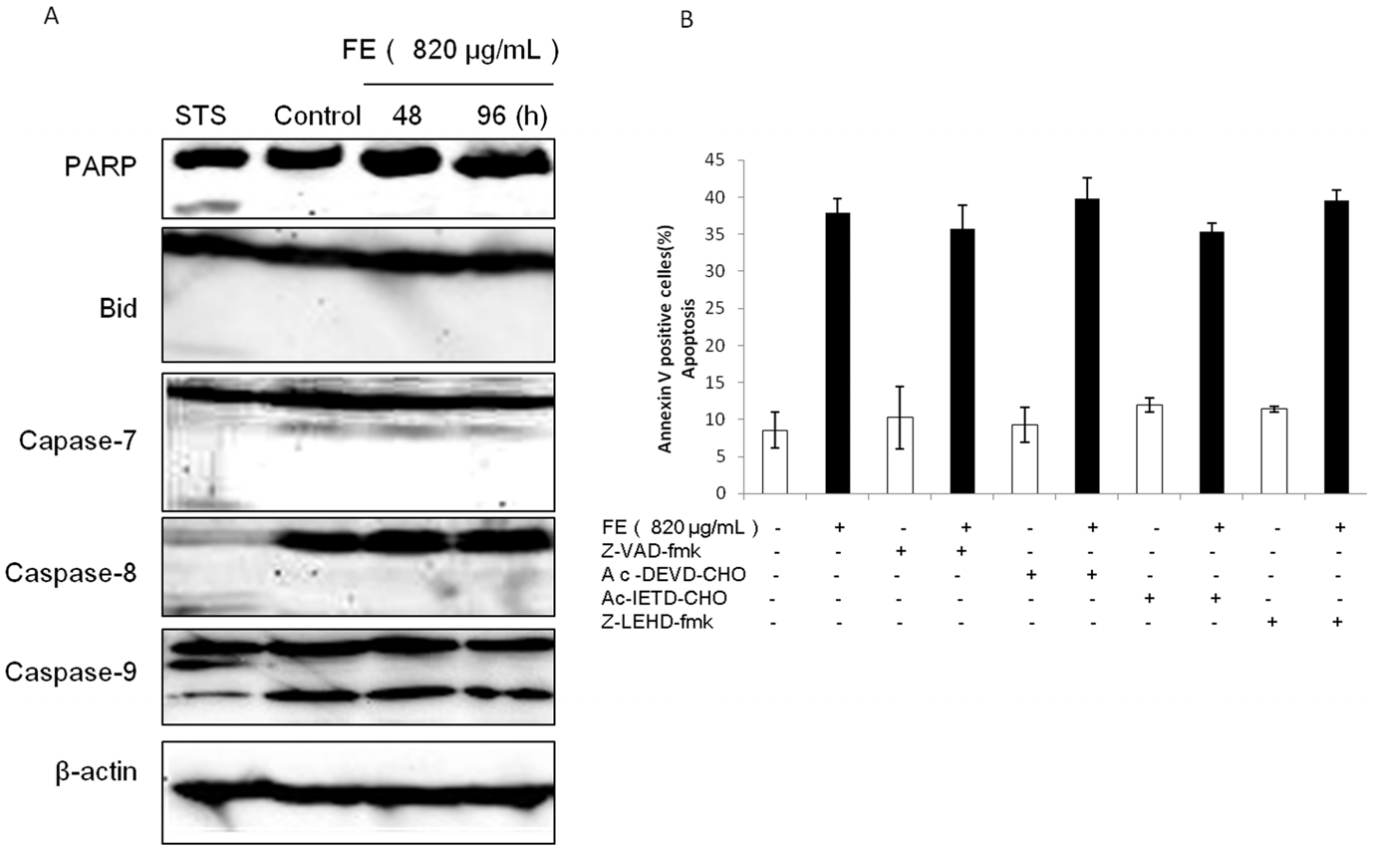
Caspase-independent apoptosis is induced by FE. (A) MCF-7 cells were treated with or without 820 µg/mL FE for different time periods. Cells treated for 6 h with 5 nM staurosporine (STS) served as a positive control. Cleavage of PARP, Bid, and caspase family members was detected using western blotting. (B) Effects of caspase inhibitors on apoptosis induced by FE. MCF-7 cells were pretreated with various caspase inhibitors at 10 µM for 1 h followed by incubation with 820 µg/mL FE for 48 h. The apoptotic cells were evaluated by annexin/PI double staining using the IN Cell Analyzer 1000. All results were obtained from 3 independent experiments.

MCF-7 cells are known to not express caspase-3 [34]. To detect whether other caspases are involved in FE-mediated apoptosis, the expression of members of the caspase family was analyzed by western blotting. In our hands, MCF-7 cells treated with FE showed no significantly increased cleavage of caspase-9 and no activation of PARP, Bid, caspase-7, or caspase-8 (Figure 3A). Furthermore, the caspase inhibition assay, which uses a general caspase inhibitor Z-VAD-fmk as well as inhibitors specific to caspase-3/7, caspase-8, and caspase-9, was performed with annexin V/PI double staining analysis. However, as shown in Figure 3B, all caspase inhibitors failed to attenuate FE-induced apoptotic cell death. Additionally, Z-VAD-fmk showed no protective effects on phosphatidylserine exposure induced by FE (Figure 2E). These results suggest that caspase activation is not necessary for induction of apoptosis by FE in MCF-7 cells.

### FE causes mitochondrial dysfunction and regulation of expression of Bcl-2 family proteins

**Figure 4:**
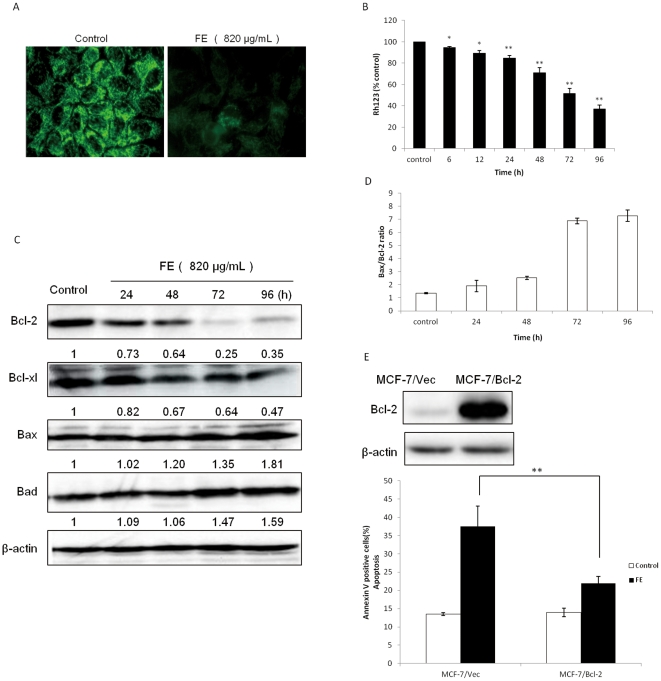
FE induces loss of ΔΨm and regulation of Bcl-2 family proteins expression. (A and B) MCF-7 cells were treated with or without 820 µg/mL FE for the indicated time periods. Disruption of ΔΨm was observed by Rh123/PI double staining and analyzed by the IN Cell Analyzer 1000. The percentage of cells retaining Rh123 in each treatment group was compared with the untreated control. (C) MCF-7 cells were treated with or without 820 µg/mL FE for different time periods. Bcl-2, Bcl-xl, Bax, and Bad were determined by western blotting using specific antibodies. (D) A densitometric analysis was used to quantify the levels of Bax and Bcl-2 to evaluate the effect of FE on the Bax/Bcl-2 ratio. (E) Bcl-2 levels in MCF-7/Vec and MCF-7/Bcl-2 cells were detected by Western blotting. MCF-7/Vec and MCF-7/Bcl-2 cells were treated with or without 820 µg/mL FE for 48 h and analyzed by Annexin V/PI assay. All of the experiments were performed at least in triplicate. All results were obtained from three independent experiments. A significant difference between treatment and control groups is indicated by p<0.05(*) or p<0.01(**).

Mitochondrial membrane potential regulates mitochondrial permeability. This plays an important role in triggering apoptotic pathways [35]. We examined the effect of FE on the ΔΨm by rhodamine 123 (Rh123) staining. As shown in Figure 4A, FE treatment of MCF-7 cells induces dissipation of ΔΨm as indicated by a decrease in green fluorescence emission. The time course analysis revealed that significant numbers of cells lose ΔΨm in FE-treated MCF-7 cells (Figure 4B). Bcl-2 family proteins have been reported to regulate MMP [36]–[37]. We therefore examined the expression of Bcl-2 family proteins in FE-treated MCF-7 cells in a time-dependent manner. Western blotting analysis revealed that FE treatment suppresses the expression of anti-apoptotic proteins such as Bcl-2 and Bcl-xl and moderately increases the expression levels of pro-apoptotic proteins such as Bax and Bad (Figure 4C). Furthermore, the ratio of Bax and Bcl-2 was measured by quantification of the bands. As shown in Figure 4D, FE treatment induces a time-dependent increase in the Bax/Bcl-2 ratio in MCF-7 cells. We then established control (MCF-7/Vec) and Bcl-2 overexpressing MCF-7 (MCF-7/Bcl-2) cell lines as detailed in Materials and Methods. MCF-7/Bcl-2 cells expressed significantly higher levels of Bcl-2 than the control cells (Figure 4E). Moreover, FE-treated MCF-7/Bcl-2 cells demonstrated consistently lower rates of apoptosis compared with those of similarly treated MCF-7/Vec cells. These data indicate that FE treatment led to mitochondrial dysfunction in MCF-7 cells.

### FE induces translocation of mitochondrial apoptotic factors

**Figure 5:**
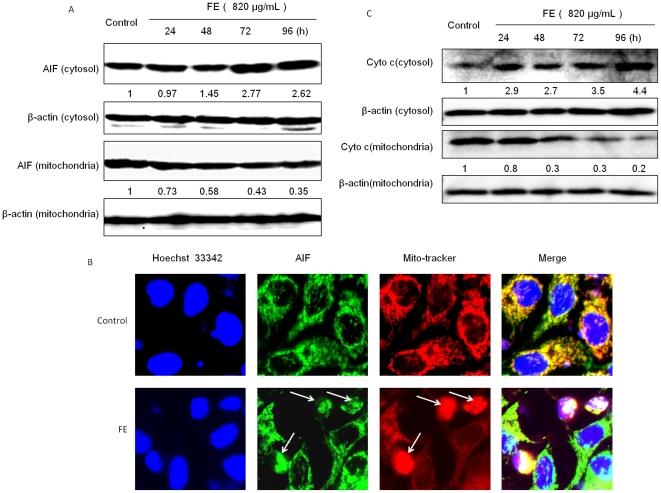
Translocation of mitochondrial apoptotic factors is induced by FE. (A) Time-dependent release of AIF from mitochondria into the cytosol was measured by western blotting analysis. (B) FE induces translocation of AIF from the mitochondria to the nucleus. MCF-7 cells were treated with 820 µg/mL FE for 96 h. After incubation with 100 nM MitoTracker Red (red fluorescence), the cells were fixed and permeabilized followed by immunostaining with an antibody specific for AIF (green fluorescence) and counterstaining with Hoechst 33342 (blue fluorescence) to visualize nuclear morphology. (C) Time-dependent release of cytochrome *c* from mitochondria into the cytosol was measured by western blotting analysis. Similar results were obtained from 3 independent experiments. Each image shown is representative of 20 random fields observed.

Mitochondrial damage occurring as a result of drug-induced apoptosis is often accompanied by the release of mitochondrial apoptotic factors into the cytosol [38]. We therefore examined whether AIF plays a role in FE-induced apoptosis. The results of western blotting showed that FE treatment induces the release of AIF from the mitochondria into the cytosol in a time-dependent manner (Figure 5A). AIF localization was determined by immunofluorescence using a specific antibody. The mitochondria and nuclei were labeled with MitoTracker and Hoechst 33342, respectively. As shown in Figure 5B, the untreated MCF-7 cells exhibit AIF staining in a punctured pattern, indicating its normal location in the mitochondria together with normal nuclear morphology. On the other hand, MCF-7 cells treated with FE exhibit increased diffuse staining of AIF in the nucleus accompanied by chromatin condensation and nuclear fragmentation. These characteristics are typically caused by apoptosis. In addition, cytochrome *c*, another apoptotic regulatory protein, was detected by western blotting. The time course analysis showed the release of cytochrome *c* from the mitochondria into the cytosol (Figure 5C).

### FE induces the activation of MAP kinases in MCF-7 cancer cells

**Figure 6:**
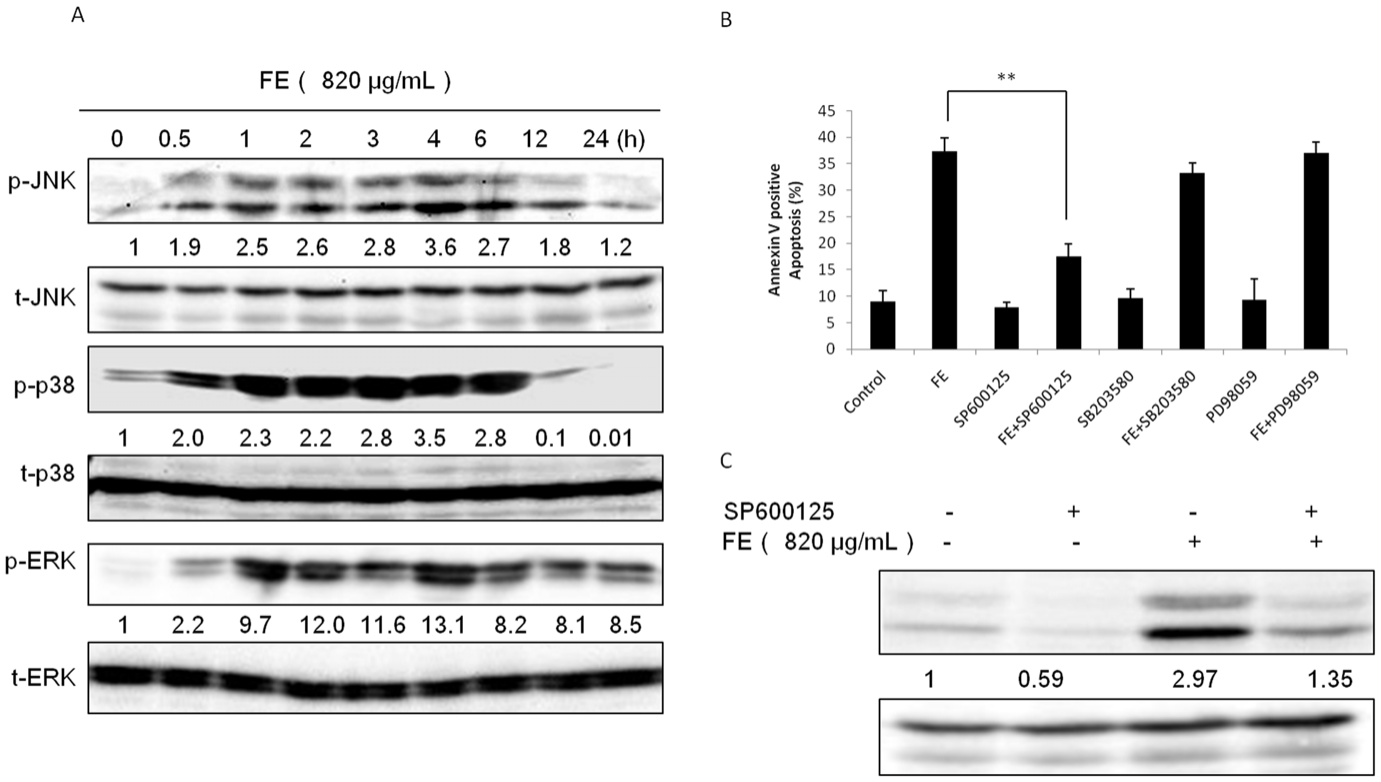
Roles of MAPKs in FE-induced apoptosis. (A) MCF-7 cells were treated with 820 µg/mL FE for the indicated time periods. JNK, p38, and ERK1/2 MAPKs and their phosphorylated forms were determined by western blotting using specific antibodies. (B) Effects of MAPK inhibitors on apoptosis induced by FE. MCF-7 cells were pretreated with 10 µM SP600125, SB203580, or PD98059 for 1 h and then exposed to 820 µg/mL FE for 48 h (controls were not exposed to FE). After the treatment, annexin V-PI double staining was used to analyze apoptotic cell death using the IN Cell Analyzer 1000. All results were obtained from 3 independent experiments. Differences with p<0.05(*) or p<0.01(**) are considered statistically significant. (C) Cells were treated for 1 h with or without 10 µM SP600125 followed by 820 µg/mL FE for 6 h. Phosphorylated forms of JNK were determined by western blotting using a specific antibody.

The MAPK family is involved in processes that induce cell death [31], [39]. In view of this evidence, the effects of FE on the phosphorylation of JNK, p38, and ERK1/2 (which correlates with activation of these proteins) were evaluated. MCF-7 cells were first treated with FE at a concentration of 820 µg/mL for different lengths of time, and phosphorylation of JNK, p38, and ERK1/2 were measured by western blot analysis. As shown in Figure 6A, the phosphorylation of JNK, p38, and ERK1/2 was detectable after as little as 30 minutes of FE treatment and persisted for at least 6 h of treatment. These results indicate that the JNK, p38, and ERK1/2 pathways were activated in response to FE in MCF-7 cancer cells. To evaluate whether these kinds of changes caused by FE are associated with apoptosis, we examined the effects of specific inhibitors on MCF-7 cancer cells in the following study. Among the different inhibitors tested (Figure 6B), we observed a significant decrease in the population of apoptotic cells in cells treated for 48 h with FE in the presence of the JNK-selective inhibitor SP600125. However, pretreatment with an inhibitor of p38 (SB203580) or an inhibitor of ERK (PD98059) had little effect. Pretreatment with SP600125 also somewhat decreased JNK phosphorylation after 6 h of FE treatment (Figure 6C).

### Effect of ROS on FE-induced apoptosis in MCF-7 cancer cells

**Figure 7:**
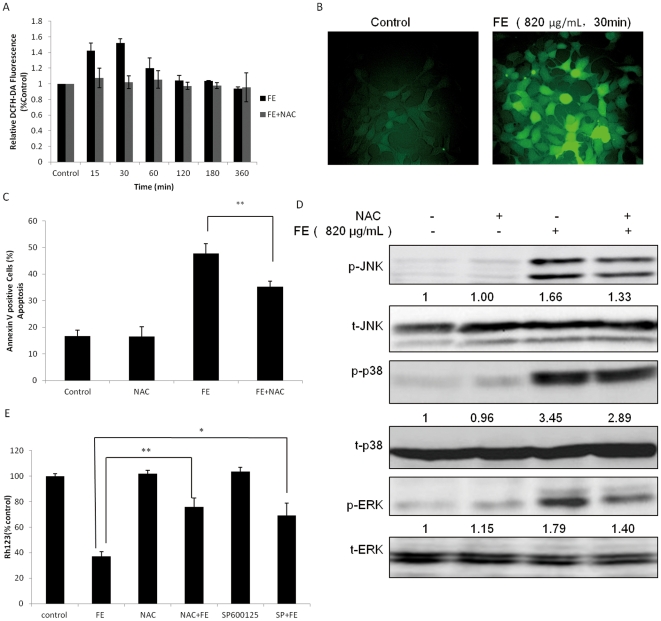
Effect of ROS on FE-induced apoptosis. (A and B) Cells were pretreated for 1 h with or without 2 mM NAC followed by 820 µg/mL FE for 15–360 min. The cells were labeled with 5 µM DCFH-DA, and the fluorescence was measured using the IN Cell Analyzer 1000 as explained in the methods section. (C) Cells were treated with 820 µg/mL FE for 48 h after incubation with 2 mM NAC for 1 h and then the apoptotic cells were analyzed using the IN Cell Analyzer 1000. (D) MCF-7 cells were treated with 2 mM NAC for 1 h before treatment with 820 µg/mL FE for 6 h. Phosphorylation of JNK, p38, and ERK1/2 MAPKs was detected by western blotting. (E) Cells were pretreated with 2 mM NAC and 10 µM SP600125 for 1 h and then incubated with 820 µg/mL FE for 48 h. Disruption of ΔΨm was detected by Rh123 staining by using the IN Cell Analyzer 1000. All results were obtained from 3 independent experiments. Differences with p<0.05(*) or p<0.01(**) are considered statistically significant.

We used the fluorescent probe DCFH-DA to measure the generation of intracellular ROS in FE-treated MCF-7 cells. As shown in Figure 7A and 7B, when the cells were treated with FE, DCFH-DA-derived fluorescence was observed to increase by 1.5-fold after the first 30 min, after which the fluorescence steadily decreased to less than the level of the control within 6 h. Moreover, the ROS generation was almost completely inhibited by pretreatment with the scavenger NAC in a time-dependent manner (Figure 7A). To determine whether ROS participates in apoptosis, we tested the effect of NAC on FE-treated MCF-7 cells and found that NAC pretreatment of cells treated with FE for 48 h partially inhibited the accumulation of apoptotic cells relative to cells treated with FE alone (Figure 7C).

Interestingly, the detailed analysis of the time course for ROS generation showed that the fluorescence started to increase at 15 min, with a peak at 30 min (Figure 7A). The phosphorylation of JNK, p38, and ERK1/2 was detected at 30 min and was sustained for at least 6 h (Figure 6A). It was important to compare this time course with the ΔΨm changes caused by FE in the MCF-7 cells (Figure 4B). In view of these results, we decided to study whether the generation of ROS could be coincident with the phosphorylation of JNK, p38, and ERK1/2 and loss of ΔΨm in FE-treated MCF-7 cancer cells. As shown in Figure 7D, pretreatment with 2 mM NAC partially attenuated the phosphorylation of JNK, p38, and ERK1/2 after 6 h of FE treatment. This indicates that ROS are required for phosphorylation of JNK, p38, and ERK1/2. Furthermore, pretreatment with either SP600125 or NAC partially inhibited the dissipation of ΔΨm after 48 h of FE treatment (Figure 7E), indicating that ROS-dependent JNK phosphorylation is an event that occurs upstream of mitochondrial membrane permeabilization in FE-treated MCF-7 cells.

### Effect of FE on cellular metabolism

**Figure 8:**
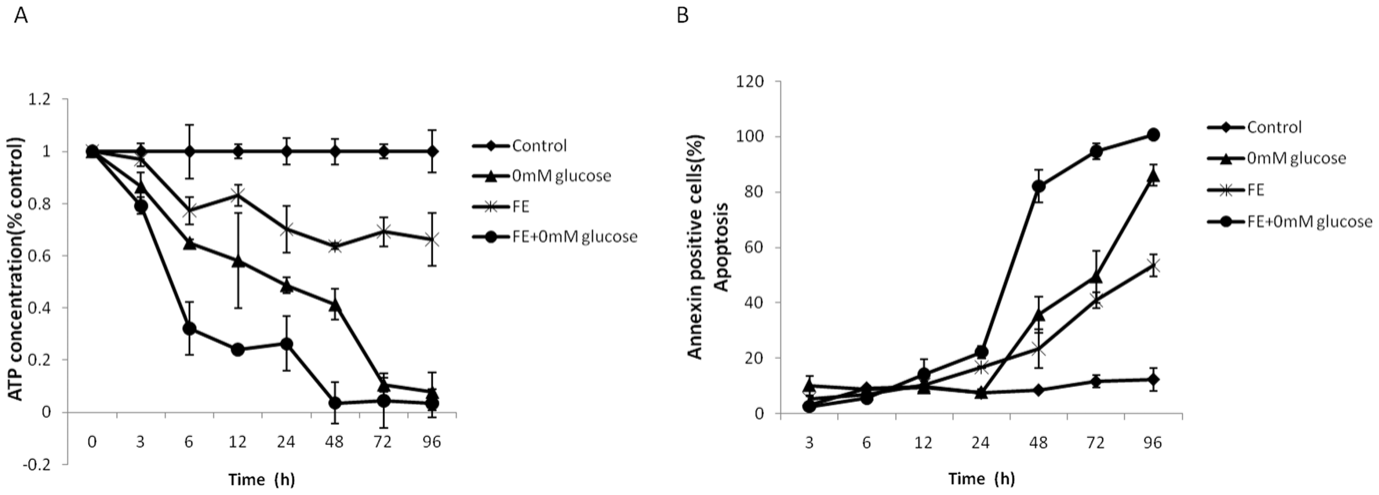
Effect of FE on the cellular metabolism. (A) Cells were treated with glucose deprivation, 820 µg/mL FE, or 820 µg/mL FE in combination with glucose deprivation. The ATP concentration was analyzed with the Bioluminescence assay Kit. Reactions were carried out in triplicate and ATP concentrations were expressed as percentages of untreated controls. (B) Cells were treated with glucose deprivation, 820 µg/mL FE, or 820 µg/mL FE in combination with glucose deprivation. Annexin V-PI double staining was used to analyze apoptotic cell death using the IN Cell Analyzer 1000.

To investigate whether FE regulates cellular metabolism, we examined cellular ATP levels in MCF-7 cells. As shown in Figure 8A, we detected a reduction in intracellular ATP levels in MCF-7 cells after 6 h of FE treatment. An even more substantial decrease in ATP levels was observed when FE was combined with glucose-free medium. In contrast, annexin V-FITC staining revealed that the induction of cell death lagged behind the reduction of ATP levels (Figure 8B). An obvious increase in annexin V-FITC staining was observed after 24 h of FE treatment irrespective of the rate of ATP depletion.

## Discussion

Fucoidan is a potent inducer of apoptosis in various cancer cell lines. However, the molecular mechanism by which fucoidan initiates apoptosis has not been well characterized. Several studies have shown that fucoidan induces extrinsic or intrinsic apoptotic signaling in different cancer cell types via alteration of expression or activities of mitochondria-associated proteins, cell cycle regulatory proteins, proteases, and transcription factors [9]–[15]. It has been reported that at a concentration of 1 mg/mL, fucoidan induced apoptosis in MCF-7 cells via a caspase-8 dependent pathway [13] and exhibited antitumor activity toward Huh7 cells via downregulation of CXCL12 expression [14]. At 3 mg/mL, fucoidan has been found to cause cell death through inactivation of the NF-κB and AP-1 pathways in human T-cell leukemia virus type1-infected T cells [10]. *In vivo*, 150 mg/kg (body weight) fucoidan inhibited the growth of tumors of a human T-cell leukemia virus type1-infected T cell line transplanted subcutaneously in severe combined immune deficient mice [10]. As these studies were based on high doses of fucoidan, the dose of fucoidan associated with pharmacologic exposure and antitumor effects *in vivo* requires further study. In this study, we noted that FE at doses between 82 µg/mL and 820 µg/mL inhibits the growth of MCF-7, MDA-MB-231, HeLa, and HT1080 cells, but is less toxic towards the human mammary epithelial cell line MCF-10A. Cell cycle distribution analysis, nuclear staining, and annexin V-PI staining were used to monitor FE-induced apoptotic cell death in MCF-7 cells. L-fucose at 1 mg/mL did not demonstrate any significant effect on MCF-7 cells by annexin V/PI assay. Moreover, we found that FE does not affect cell cycle progression. This precludes the possibility that FE-dependent cytotoxicity in MCF-7 cells is due to cell cycle arrest.

To study the mechanisms by which FE treatment induces apoptosis in MCF-7 cells, we examined a number of markers that are associated with apoptotic cell death. Previous studies have shown that caspases act as key executors of fucoidan-induced apoptosis [8]–[15]. Although FE treatment causes activation of caspase-9, specific inhibitors failed to attenuate FE-induced apoptotic cell death in MCF-7 cells. These results have led us to propose a model in which FE induces caspase-independent apoptosis in MCF-7 cancer cells.

Mitochondria have been shown to play a central role in the apoptotic process. Both the extrinsic pathway and the intrinsic pathway can converge at the mitochondrial level and trigger mitochondrial membrane permeabilization [35], [40]–[41]. In our study, depletion of ΔΨm in MCF-7 cancer cells was detected after treatment with FE for 6 h (Figure 4B). This is consistent with the hypothesis that mitochondrial dysfunction is an early event during the process of apoptosis [35]. Any imbalance of the expression levels of anti- and pro-apoptotic Bcl-2 family proteins will disrupt the integrity of the outer mitochondrial membrane [36]–[37]. We observed that FE up-regulates the expression of pro-apoptotic proteins Bax and Bad, and down-regulates the expression of anti-apoptotic proteins Bcl-2 and Bcl-xl. The Bax/Bcl-2 ratio, which helps to determine the susceptibility of cells to a death signal by regulating the function of mitochondria [37], was found to increase in a time-dependent manner. Importantly, Bcl-2 overexpression partially protected MCF-7 cells against FE toxicity, which further indicates the involvement of mitochondria in FE-regulated cell death. Membrane depolarization (loss of ΔΨm) induces mitochondrial permeability transition pore (PTP) formation [42]. It is known that cytochrome *c* is released, along with AIF and Smac/Diablo, from the inter-membrane space of mitochondria into the cytosol through the mitochondrial PTP [38], [42]. The data presented in the present study indicate that FE treatment induces the migration of cytochrome *c* from the mitochondria to the cytosol. We also found that AIF is translocated from the mitochondria to the nucleus, where it causes chromatin condensation and large-scale DNA fragmentation. Interestingly, AIF has been shown to be a critical mediator of a caspase 3-independent apoptosis pathway in several cell lines [20], [22]. Further genetic targeting experiments will be required to determine if AIF is required and is sufficient to promote FE-induced apoptosis. Collectively, these observations indicate an intriguing role for FE in provoking mitochondrial damage and regulating apoptotic proteins at or in mitochondria in MCF-7 cells. Thus, increased mitochondrial depolarization, regulation of Bcl-2 proteins, and release of cytochrome *c* and AIF appear to be major steps leading to the induction of apoptosis in FE-treated MCF-7 cells.

It has been reported that MAP kinases are involved in activation of fucoidan-induced apoptosis [8], [11]. We demonstrated that phosphorylation of JNK, p38, and ERK1/2 occurred rapidly within 30 min after FE exposure in MCF-7 cancer cells. p38 kinase has been demonstrated to be an essential regulator of sustained G2 arrest [30], [43]. However, we found that FE does not affect cell cycle progression and that the inhibition of p38 failed to block apoptosis, suggesting that the involvement of p38 is not critical for FE-induced cell death. Fucoidan has been found to induce MEKK1-dependent and Raf-independent activation of MEK1/2-ERK1/2 in leukemic cells, and this activation in turn affects the activation of JNK [44]. Although our results showed that FE induced phosphorylation of ERK 1/2 in MCF-7 cells, inhibition of ERK 1/2 also failed to block apoptosis. This difference in the effect of FE on ERK 1/2 may be due to cell type-specific factors or to the diversity of fucoidan structures and their subsequent signaling transduction pathways. A previous study demonstrated that the duration and intensity of JNK activation are associated with cell death [45]. We demonstrated that JNK phosphorylation occurred rapidly, within 30 min of FE treatment, and persisted for at least 6 h after FE exposure; we also observed increased levels of both phosphor-JNK1 and phosphor-JNK 2 isoforms. Apoptotic cell death is significantly promoted in cells expressing JNK but effectively suppressed in cells expressing a dominant-negative (dn) JNK 1 mutant [46]. Moreover, dnJNK1 has been reported to block the anti-IgM-induced ΔΨm in B cells [47]. In our study, JNK1 phosphorylation was efficiently inhibited by SP600125, a specific inhibitor of JNK, and this perturbation of JNK phosphorylation prevented apoptosis. Furthermore, SP600125 attenuated the loss of ΔΨm, suggesting that phosphorylation of JNK was partially responsible for the collapse of ΔΨm. These results suggest that JNK acts as an important mediator of the pro-apoptotic effects of FE. JNK is likely to phosphorylate and activate a mitochondrial target in a process directly or indirectly linked to cell death in FE-treated MCF-7 cells. It is possible that sustained phosphorylation of JNK perturbs the balance of Bcl-2 family proteins to commit the cell to apoptosis. The fact that Jnk^-/-^ MEFs exhibit defects in the release of mitochondrial cytochrome *c* is further evidence for a JNK-stimulated apoptosis pathway [48]. In addition, it has been reported that TNFα-dependent ROS production causes sustained JNK activation and cell death [49]. Indeed, a mere transient increase in ROS is linked to opening of the mitochondrial permeability transition pore, mitochondrial translocation of Bax and Bad, and cytochrome *c* release [33]. Our studies show that FE induced generation of ROS. The scavenger NAC was found to decrease the percentage of cells that lose ΔΨm and inhibit apoptotic cell death. Furthermore, pretreatment with NAC moderately decreased the FE-induced phosphorylation of JNK, p38, and ERK1/2 in MCF-7 cells, suggesting that an ROS signaling pathway is related to the phosphorylation of JNK, p38, and ERK1/2. These results support the hypothesis that the generation of ROS precedes the phosphorylation of JNK, p38, and ERK1/2 and that this generation of ROS could cause the mitochondrial permeability transition.

Cancer cells exhibit increased glycolysis and use this metabolic pathway to ATP as a main source of their energy supply [50]. Our results showed that ATP depletion occurred in FE-treated MCF-7 cells and that this ATP depletion preceded changes in Annexin V-FITC staining. Glucose deprivation is known to result in depletion of cellular ATP and in turn cause cell death [51]–[52]. Moreover, cellular glucose uptake is related to the sensitivity of the drug [53]–[55]; the reduction in ATP depletion might reflect the sensitivity of FE in MCF-7 cells. It is also possible that the decreased ATP is attributable to the cell death induced by FE, as plasma membrane damage renders ATP completely undetectable [56]. Together, these data suggest that FE impacts intracellular ATP concentration. Future studies focused on this observation will improve our understanding of the cellular metabolic signals that induce and effect FE-mediated cell death.

Fucoidan extract has been used in clinical trials in recent years. Nishimoto observed rapid increases of interleukin 12, interferon γ, and TNF-α values in the blood of a breast cancer patient (45-years-old female) after oral administration of 200 mL fucoidan extract solution (40 mg/kg/day) for 1.5 and 4 months [4]. Two cervical cancer patients (a 49-year-old female and a 24-year-old female), a renal cancer patient (81-year-old male), and a liver cancer patient (74-year-old female) also experienced improvement in their symptoms after receiving fucoidan extract orally at 30–40 mg/kg/day [4]. Since 2004, Takada [57] has performed a clinical trial in three patients (a 47-year-old female, a 78-year-old male, and an 84-year-old female) with hepatocellular carcinoma. The patients who received 180–400 mL of FE orally per day (30–80 mg/kg/day) showed overall decreases in their α-fetoprotein (AFP) and des-y-carboxyprothrombin (DCP) levels after several days' treatment. The patients experienced significant quality-of-life improvements during the initial 3–5 months of oral therapy and subsequent remission or disappearance of their cancers. Although there is no clearly evident theoretical mechanism to account for the potentially improved response and survival rates in carcinoma patients treated with FE, these findings suggest that FE is safe for cancer patients and has potential for use for cancer prevention and intervention in humans. Fucoidan extract of an average molecular weight of 28.8 kDa was detected without degradation in subjects' serum, plasma, and urine 6 h after oral administration [58]. In fact, the average molecular weight of FE used in our study was about 500 Da, far lower than that of traditional fucoidan. Sulfated polysaccharides from the same family may exhibit different properties depending on their electrical charges, degrees of sulfation, and molecular weights [1], [59]–[60]. The low molecular weight of FE is expected to facilitate rapid absorption into blood vessels and effective action against cancers of various organs.

**Figure 9:**
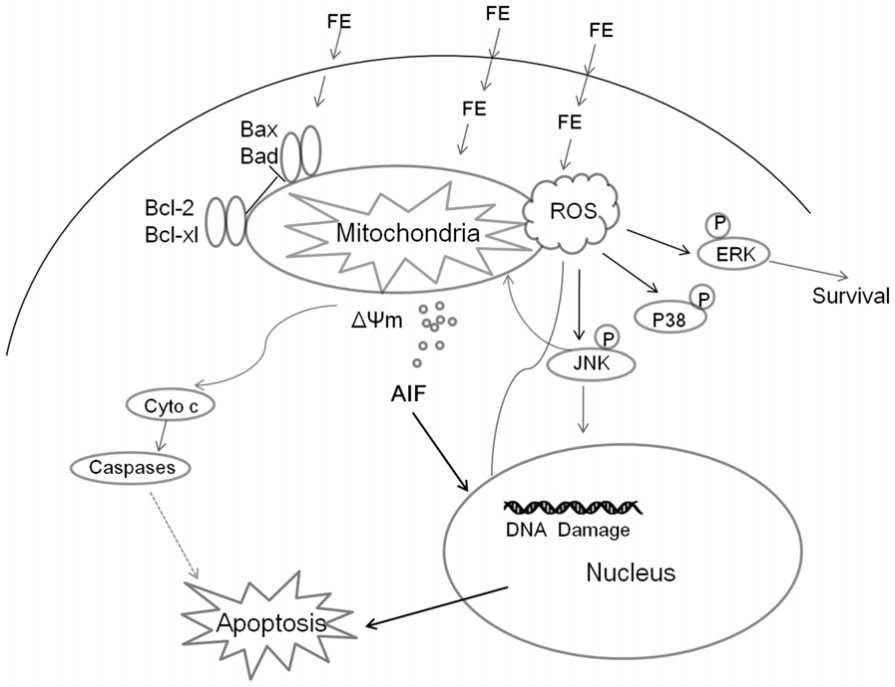
A model summarizing the mechanism of FE-induced apoptotic cell death in MCF-7 breast cancer cells.

In conclusion, our study demonstrates that FE inhibits growth of the MCF-7, MDA-MB-231, HeLa, and HT1080 cell lines. We present evidence that FE induces a caspase-independent mitochondria-mediated apoptotic pathway in MCF-7 cells (Figure 9). FE treatment increases phosphorylation of JNK, p38, and ERK1/2 kinases and triggers MMP by modulating expression of Bcl-2 family members and depolarizing mitochondrial membranes; these effects may be due in part to increased levels of ROS. Upstream activation of JNK is a functionally relevant event that promotes ΔΨm dissipation and subsequent apoptosis. MMP is followed by the release of cytochrome *c* and AIF. AIF translocates into the nucleus and induces chromatin condensation and DNA fragmentation, leading to nuclear apoptosis. These findings greatly contribute to the understanding of the anti-tumor activity of FE. Combination treatment with FE for cancer is already under investigation; positive results, if confirmed, would provide invaluable insight into new approaches to the development of effective chemotherapy regimens.

## Materials and Methods

### Materials

The abalone glycosidase-digested fucoidan extract prepared from seaweed Mozuku of *Cladosiphon novae-caledoniae* Kylin from the Kingdom of Tonga, which is commercially available as a product named “Power fucoidan” and was donated for this study by the Daiichi Sangyo Corporation (Osaka, Japan).

3-(4,5-Dimethyl-2-thiazolyl)-2,5-diphenyl-*2H*-tetrazolium bromide (MTT) and propidium iodide (PI) were purchased from Wako Pure Chemical Industries, Ltd. (Osaka, Japan). The annexin V-FITC apoptosis kit was obtained from Medical and Biological Laboratories Co., Ltd. (Nagoya, Japan). Rh123 and -*Cellstain*®- Hoechst 33342 were obtained from DOJINDO Laboratories (Kumamoto, Japan). 2′,7′-Dichlorofluorescin diacetate (DCFH-DA) was purchased from Merck KGaA (Darmstadt, Germany). The Mitochondrial/Cytosol Fractionation Kit was obtained from BioVision (San Francisco, CA). MitoTracker Red CMXRos Mitochondrial Probe was purchased from Molecular Probes, Inc. (Eugene, OR). The general inhibitor (Z-VAD-fmk) and the caspase-3/7 inhibitor (Ac-DEVD-CHO) were purchased from Promega (Madison, WI). The caspase-8 inhibitor (Ac-IETD-CHO) and the caspase-9 inhibitor (Z-LEHD-fmk) were purchased from BD Biosciences (San Diego, CA). The JNK inhibitor SP600125 and the p38 inhibitor SB203580 were purchased from Enzo Life Sciences International, Inc. (Plymouth Meeting, PA). Staurosporine (STS) and the ROS inhibitor NAC were purchased from Sigma-Aldrich Co. (St. Louis, MO). The ERK inhibitor PD98059 (Merck KGaA) was used in this study. All of the primary antibodies used in the study were purchased from Cell Signaling Technology, Inc. (Danvers, MA). Horseradish peroxidase (HRP)-linked anti-rabbit/mouse (Cell Signaling Technology) or Alexa-488-labeled goat anti-rabbit (Invitrogen, Carlsbad, CA) IgG antibodies were used as secondary antibodies.

### Cell culture

The human breast cancer cell lines (MCF-7 and MDA-MB-231), HeLa cervical carcinoma cell line, HT1080 fibrosarcoma cell line, and human mammary epithelial cell line (MCF-10A) were obtained from the American Type Culture Collection (Manassas, VA). All cell lines except for MCF-10A were maintained in DME medium (1 mg/mL glucose) supplemented with 10% fetal bovine serum in an incubator in a humidified atmosphere of 5% CO_2_, at 37°C. MCF-10A cells were cultured in mammary epithelial basal medium (Lonza Group Ltd., Basel, Switzerland). After 24 h, the medium was replaced with fresh medium containing various concentrations of FE. Cells grown in a medium containing phosphate-buffered saline (PBS) without FE served as a control.

### Cell proliferation assay

Cell proliferation was analyzed using the MTT assay. Cells were seeded in 96-well plates at densities of 1000 to 3000 cells/well (1000 cells/well of malignant cell lines, 3000 cells/well of non-malignant MCF-10A) and incubated for different time periods with or without different concentrations of FE. At the required time point, 10 µL of MTT solution (5 mg/mL) was added to each well. The plates were incubated for an additional 4 h at 37°C. The medium was removed and 200 µL DMSO was added to each well and pipette up and down several times to dissolve the formazan. The absorbance of each well was measured at 570 nm with a microplate reader (Tecan Group Ltd., Männedorf, Switzerland).

### Flow cytometric analysis

Evaluation of the cell cycle distribution and sub-G1 peaks by flow cytometric analysis was performed as follows: cells were collected by trypsin, washed in cold PBS, and fixed with 70% ethanol on ice for 30 min. After removing the ethanol by washing with PBS, the cells were incubated in 10 µg/mL RNase A for 30 min at room temperature and then in 20 µg/mL PI for 10 min in the dark. Cells were then analyzed by flow cytometry (Beckman Coulter, Inc., Brea, CA). For each sample, 1×10^4^ cells were recorded.

### Apoptosis assay

The annexin V-PI double-staining method was used. MCF-7 cells were seeded onto 96-well plates (5×10^3^ cells/well) and cultured for 24 h. After treatment with or without FE for varying lengths of time, the cells were stained with the annexin V-FITC labeling solution (annexin V-fluorescein in a binding buffer containing PI and Hoechst 33342). The plates were further incubated for 15 min in the dark, and then images of the cells were acquired using an IN Cell Analyzer 1000 (GE Healthcare UK Ltd., Buckinghamshire, UK). The cells with apoptotic morphology of nuclei (condensation/fragmentation) or annexin V-positive cells were analyzed using the Developer Toolbox software (GE Healthcare). For each analysis, 3,000 cells were recorded.

### Mitochondrial membrane potential assay

Mitochondrial membrane potential was measured with the IN Cell Analyzer 1000 using Rh123, PI, and Hoechst 33342 staining. Disruption of ΔΨm is associated with a lack of Rh123 retention and a decrease in fluorescence. Briefly, after FE treatment, MCF-7 cells were washed twice with PBS and incubated with 1 µM Rh123 and 2 µg/mL Hoechst 33342 at 37°C for 30 min. Cells were then washed twice with PBS, and stained with 10 µg/mL PI for 5 min. The fluorescence of Rh123 was determined with the IN Cell Analyzer 1000. For each analysis, 3,000 cells were recorded. Cells with reduced fluorescence (less Rh123) were identified as having lost some of their mitochondrial membrane potential. Image analysis was carried out using IN Cell Investigator software with either the Developer Toolbox or the Multi-Target Analysis (MTA) Module (GE Healthcare).

### Measurement of intracellular ROS

The intracellular generation of ROS was analyzed with the IN Cell Analyzer 1000. After incubation of FE for the indicated times, MCF-7 cells were incubated with 5 µM DCFH-DA at 37°C for 30 min. Then, the cells were washed twice with PBS and stained with 2 µg/mL Hoechst 33342 at 37°C for 15 min. The cellular fluorescence intensity was measured with the IN Cell Analyzer 1000 after washing the cells twice with PBS. For each sample, 3,000 events were recorded. Image analysis was carried out using the IN Cell Investigator software, using either the Developer Toolbox or the Multi-Target Analysis (MTA) Module (GE Healthcare).

### Immunofluorescence

AIF translocation from the mitochondria to the nuclei was analyzed by immunofluorescence. Briefly, cells were incubated with different concentrations of FE. Then, the cells were incubated with the mitochondrial dye MitoTracker Red CMXRos at 200 nM for 30 min, washed with PBS twice, and fixed with 3.7% formaldehyde in PBS for 15 min. After rinsing several times with PBS, the cells were permeabilized with 0.1% Triton X-100 in PBS at room temperature for 10 min. Cells were then blocked with 0.1% BSA at room temperature for 1 h and subsequently incubated with a primary AIF antibody (1∶200) overnight at 4°C and an Alexa-488-labeled anti-rabbit IgG antibody (1∶250) for 1 h at room temperature. Nuclei were stained with Hoechst 33342 for 15 min after secondary antibody incubation. Images were then captured with the IN Cell Analyzer 1000 (GE Healthcare) with 475/535 nm excitation/emission filters.

### Western blot analysis

Cells were washed twice with cold PBS and lysed with lysis buffer (50 mM Tris-HCl, 150 mM NaCl, 2 mM EDTA, 10% Nonidet P-40, 50 mM NaF, 1 mM Na_3_VO_4_, and protease inhibitor cocktail) for 20 min on ice. Protein extracts were centrifuged at 17,530 × *g* for 10 min at 4°C. Mitochondria and cytosolic fractions were separated using the Mitochondria/Cytosol fractionation kit according to the manufacturer's protocol (BioVision). The protein concentration was determined using the Bradford Protein Assay Reagent. Equal amounts of protein from each sample were separated by electrophoresis through SDS-PAGE gels and transferred to PVDF membranes (GE Healthcare). Membranes were blocked for 1 h at room temperature in Tris-buffered saline containing 0.1% Tween-20 and 5% nonfat dry milk. The membrane was incubated overnight at 4°C with the primary antibody diluted in 5% nonfat dry milk or 5% BSA. The membranes were washed 3 times and incubated for 1 h at room temperature with the HRP-conjugated secondary antibody. Protein bands were visualized using enhanced chemiluminescence as described by the supplier (GE Healthcare).

### Cell transfection

MCF-7 cells were seeded at 2×10^5^ cells per well in 6-well plates 24 h before transfection. Transfection was performed using HilyMax (Dojindo, Kumamoto, Japan) according to the manufacturer's protocol. The MCF-7/Bcl-2 cell line overexpressing Bcl-2 was established by transfecting MCF-7 cells with the pCMV-Bcl-2 plasmid encoding human Bcl-2 cDNA followed by selection with 1 mg/mL G418 for two weeks. G418-resistant clones were pooled for further experiments. MCF-7 cells transfected with an empty pRc/CMV vector (MCF-7/vec) were used as controls.

### ATP concentration Assay

The ATP concentration was measured by the Bioluminescence assay Kit CLS II (Roche Diagnostics Co., Indianapolis, IN) according to the manufacturer's instructions. In brief, untreated or treated MCF-7 cells were collected in minimal volumes and mixed with 9 volumes of boiling 100 mM Tris, 4 mM EDTA (pH 7.75). The samples were incubated for another 2 min at 100°C and centrifuged at 1000 × *g* for 60 s. The ATP concentration was then measured with a luminometer (Berthold Technologies) against an ATP standard curve. Reactions were carried out in triplicate and ATP concentrations were expressed as percentages of untreated controls.

### Statistical analysis

All experiments were performed at least in triplicate. Data are presented as mean ± S.D., and p values were analyzed using the Student's *t*-test accompanied by analysis of variance (ANOVA) where appropriate.
